# Customized Finite Element Modelling of the Human Cornea

**DOI:** 10.1371/journal.pone.0130426

**Published:** 2015-06-22

**Authors:** Irene Simonini, Anna Pandolfi

**Affiliations:** 1 Dipartimento di Matematica, Politecnico di Milano, Milano, Italy; 2 Dipartimento di Ingegneria Civile ed Ambientale, Politecnico di Milano, Milano, Italy; Cedars-Sinai Medical Center; UCLA School of Medicine, UNITED STATES

## Abstract

**Aim:**

To construct patient-specific solid models of human cornea from ocular topographer data, to increase the accuracy of the biomechanical and optical estimate of the changes in refractive power and stress caused by photorefractive keratectomy (PRK).

**Method:**

Corneal elevation maps of five human eyes were taken with a rotating Scheimpflug camera combined with a Placido disk before and after refractive surgery. Patient-specific solid models were created and discretized in finite elements to estimate the corneal strain and stress fields in preoperative and postoperative configurations and derive the refractive parameters of the cornea.

**Results:**

Patient-specific geometrical models of the cornea allow for the creation of personalized refractive maps at different levels of IOP. Thinned postoperative corneas show a higher stress gradient across the thickness and higher sensitivity of all geometrical and refractive parameters to the fluctuation of the IOP.

**Conclusion:**

Patient-specific numerical models of the cornea can provide accurate quantitative information on the refractive properties of the cornea under different levels of IOP and describe the change of the stress state of the cornea due to refractive surgery (PRK). Patient-specific models can be used as indicators of feasibility before performing the surgery.

## Introduction

The growing demand for permanent refractive corrections and laser surgical procedures, such as PRK and LASIK, has increased the importance of corneal topography data in both clinical and research settings.

Within the past 10 years, corneal topographers have been converted from elaborate and costly devices used exclusively for clinical research to in-office tools that optometrists use daily. Along with advances in computerization and software development, topographers have become smaller, more compact, multifunctional, easy-to-handle, less expensive and more precise [1].

Modern topographers use advanced techniques which do not require anaesthetics nor direct contact with the cornea. For example, they combine Scheimpflug camera with a Placido disk [2], the ultrasound system [3], and other various different techniques (for example tomography) [1] or air puff test [4].

As an example, a combination between a rotating Scheimpflug camera and Placido disk is found in the Sirius topographer (CSO, Scandicci, Italy), a high precision system for the tridimensional imaging of both cornea and anterior segment. Merging data from Placido's rings [5] with the ones obtained at the same time from two Scheimpflug cameras [6], the topographer acquires good measurement of elevations, curvature, power, and thickness of the whole cornea [7]. Besides the images, the software associated to the topographer supplies the coordinates of points lying on the anterior surface of the cornea and the corneal thickness at the same points. In the particular case of Sirius topographer, points are collected on concentric circles and meridians. By means of mathematical interpolation of these points, it is possible to reconstruct the accurate geometry of the anterior and posterior corneal surfaces. Surface data can be acquired in preoperative and postoperative conditions, allowing for modelling the geometrical and optical changes of the cornea due to refractive intervention.

The current ophthalmologic literature testifies a growing interest in numerical models of the anterior segment of the eye. The very first contributions worked on idealized geometries and isotropic materials and addressed the simulation of refractive procedures [8–13]. Successive works included the underlying anisotropic microstructure of the stroma [14–17] and tried to focus on the qualitative evaluation of refractive surgery outcomes [18–21], or to explain the reliability of intraocular pressure measurements [22–24]. If the eye’s geometry is known and sufficient information on the material properties is available, it is possible to create a personalized numerical model of the eye that possesses predictive (therefore quantitative) abilities. Examples of numerical applications that evaluate the qualitative mechanical response of the anterior chamber of the eye to different actions can be found in the recent literature [25–27].

In view of obtaining a predictive numerical model of the anterior segment of the eye, it is necessary to use patient-specific geometry and patient-specific material models. Modern diagnostic instruments for ophthalmology acquire the tridimensional geometry of the whole anterior chamber of eye. Therefore, customized purely geometrical models are already available and standard stress analysis codes have the potential to predict the qualitative response to refractive surgery [8–11]. Regrettably, patient-specific material models are not available yet, given the difficulty to identify the material properties of the different parts of the anterior segment of the eye by means of non destructive in-vivo tests. Research is moving fast in view of tackling this missing point, trying to combine innovative experimental methods with inverse analysis in advanced identification procedures [28–30]. A promising testing procedure is the air-puff tonometry, combined with imaging and numerical analysis, but the technique is not fully developed yet [31–33] and needs to be coupled to different material models in order to reproduce the behaviour of individual corneas.

The present study has been carried out in order to validate the performance of an advanced numerical procedure that, starting from images of the anterior chamber of the eye, builds an accurate, patient-specific geometrical model of the human cornea. The geometrical model is then used in a static stress analysis solver that estimates, by means of a simplified identification procedure, the material properties of the eye according to the chosen material model [27]. The innovation of the present approach is represented by a patient-specific geometry and by a more accurate estimation of the individual material properties with respect to previous contributions [17, 20, 27, 34]. The patient-specific geometry is obtained through a sophisticated interpolation procedure on the surface points provided by the topographer.

Recent studies [35, 36] discuss including the description of the fibril organization in the human cornea. The current version of our finite element code uses improved models of statistically distributed fiber materials [37], applied over a particular organization of the collagen fibrils in the cornea [20, 34]. The material model used in the present calculations has been proved to be sufficiently accurate, robust, and efficient in previous applications [27]. For the sake of convenience, we used our model of the fibril distribution and a material model developed in our group because of the awareness of the features of the code and the large variety of output data.

## Methods

This study comprised five patients in the age range 25 to 43 in whom PRK for myopic or myopic compound astigmatism was performed between 2013 and 2014. Clinical data used in this retrospective study were collected according to a protocol approved by the Italian Data Protection Authority, and according to the principles expressed in the Declaration of Helsinki. Data were anonymized and de-identified prior to the transmission to the authors. All patients returned for a 3-month follow-up. Preoperative and short term postoperative corneal topographic data were available from topographer’s measurements and were used to set up patient-specific numerical simulations. The geometry of the cornea was provided in terms of cylindrical coordinates of the anterior corneal surface and corresponding thickness in discrete points belonging to 30 concentric circles with increasing radius of 0.2 mm and to 128 meridians.

### Patient-specific geometrical model of the cornea

The realization of a customized model of the cornea requires three steps: (i) the acquisition of a cloud of points belonging to the anterior surface of the cornea and the completion of the eventually missing points; (ii) the reconstruction of the posterior surface and of the limbus through the thickness data; (iii) the discretization into finite elements.

#### Acquisition and completion of anterior surface points

**Fig 1:**
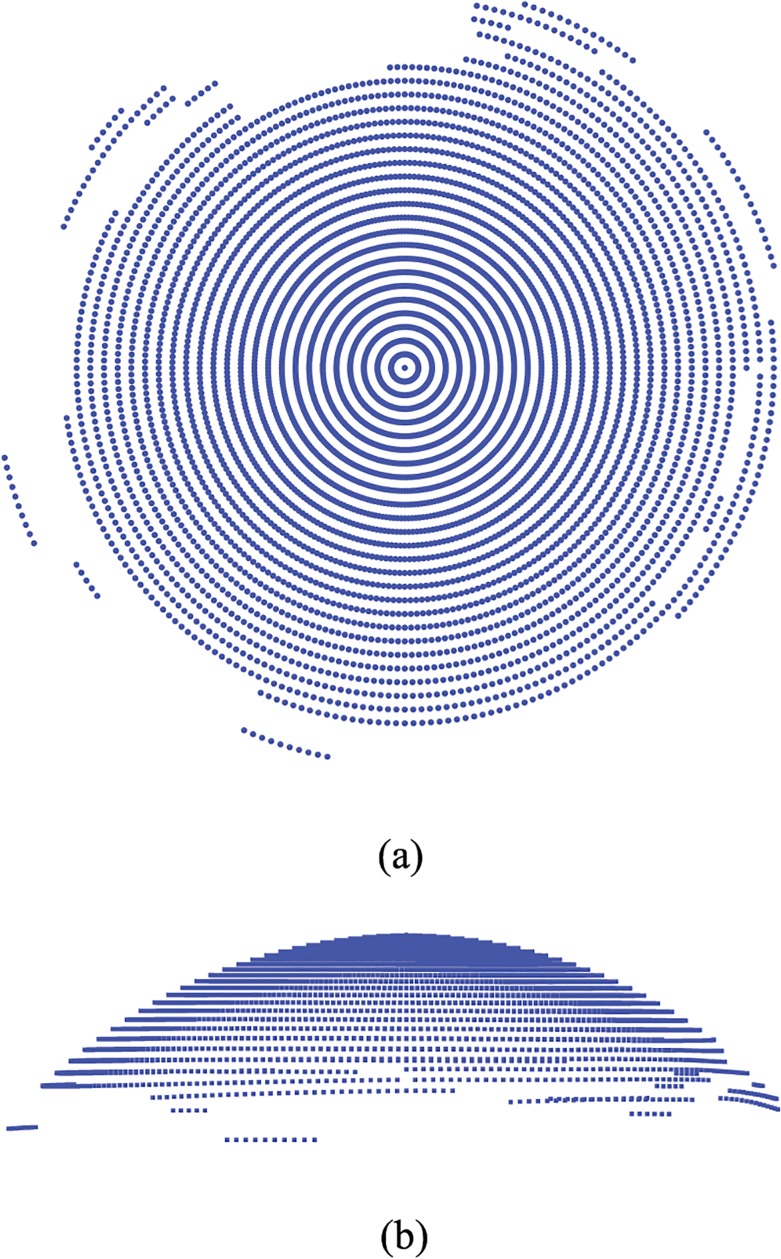
Example of original data set extracted from the topographer. Each point is visualized with a small spherical symbol. (a) Anterior surface view; (b) Nasal-temporal (NT) view.

**Fig 2:**
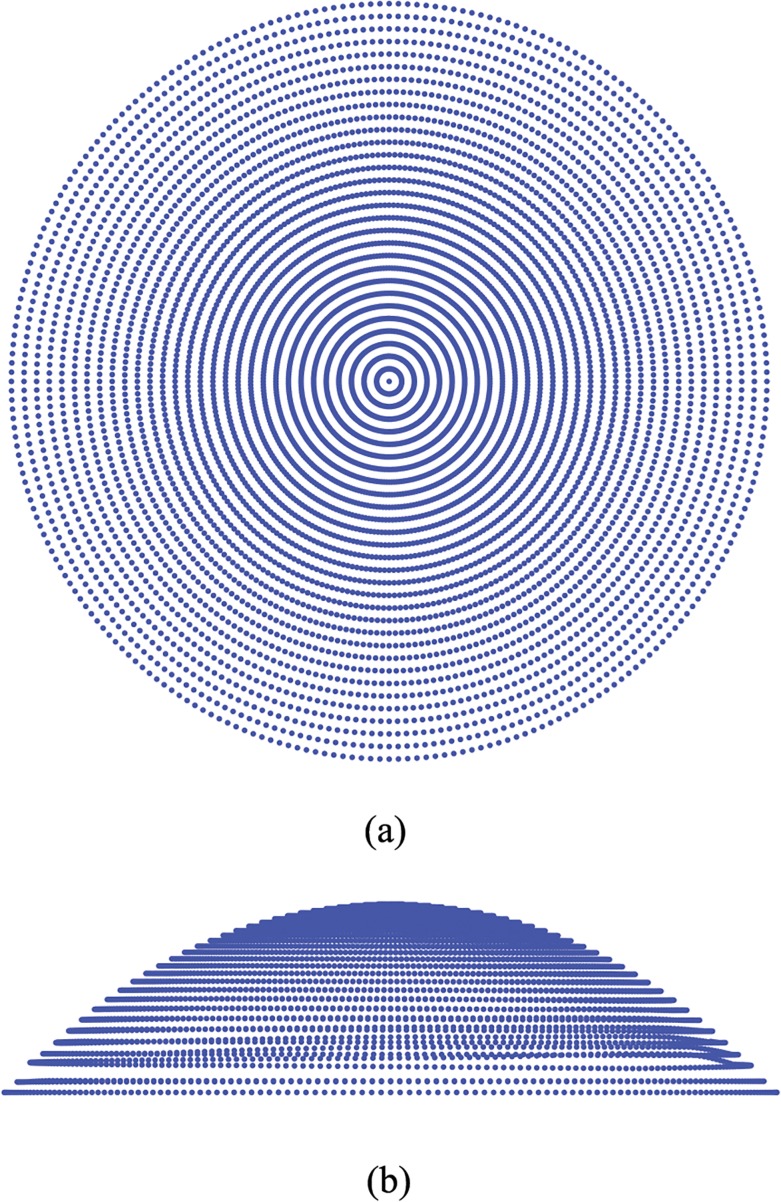
Example of full data set showing how the originally gapped data provided by the topographer have been interpolated over existing data. (a) Anterior surface view; (b) Nasal-temporal (NT) view.

**Fig 3:**
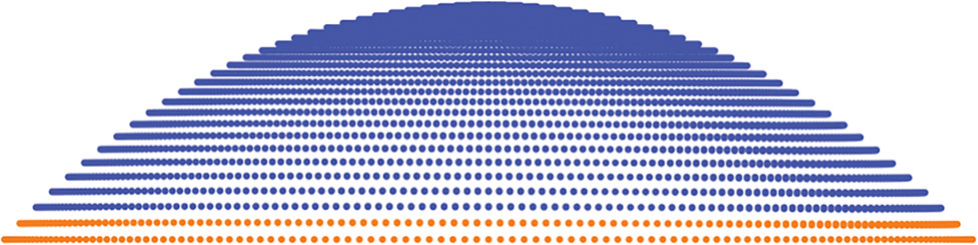
Example of extra circumferences added to reach the horizontal visible iris diameter (HVID).

The software associated to the Sirius corneal topographer provides the Cartesian coordinates of points belonging to the anterior surface of the cornea, located on concentric circumferences and meridians of the cornea. Data are exportable in different formats, and in general readable by standard commercial software. In general, a topographer’s performance in acquiring data is strongly dependent on some patient’s characteristics and external factors might affect the measurement process [7]. In particular, the presence of eyelashes or involuntary ocular movements may interfere with the data acquisition; the thickness and the stability of the tear film may also alter the image. Thus in some cases the sequence of data is characterized by gaps located in the periphery of the cornea, close to the limbus (Fig 1). When gaps are present, the cloud of points has to be completed by means of interpolation between existing values. To obtain a smooth filling of the gaps, the best choice is to adopt a linear interpolation between points belonging to the same circumference and a quadratic interpolation between points belonging to adjacent circumferences (Fig 2). In some cases, whole circles of points are missing and circumferences need to be added in order to fully cover the horizontal visible iris diameter (HVID), also called white to white distance (WTW). The additional circumferences are added by using quadratic interpolation of existing data (Fig 3).

### Construction of the posterior corneal surface

**Fig 4:**
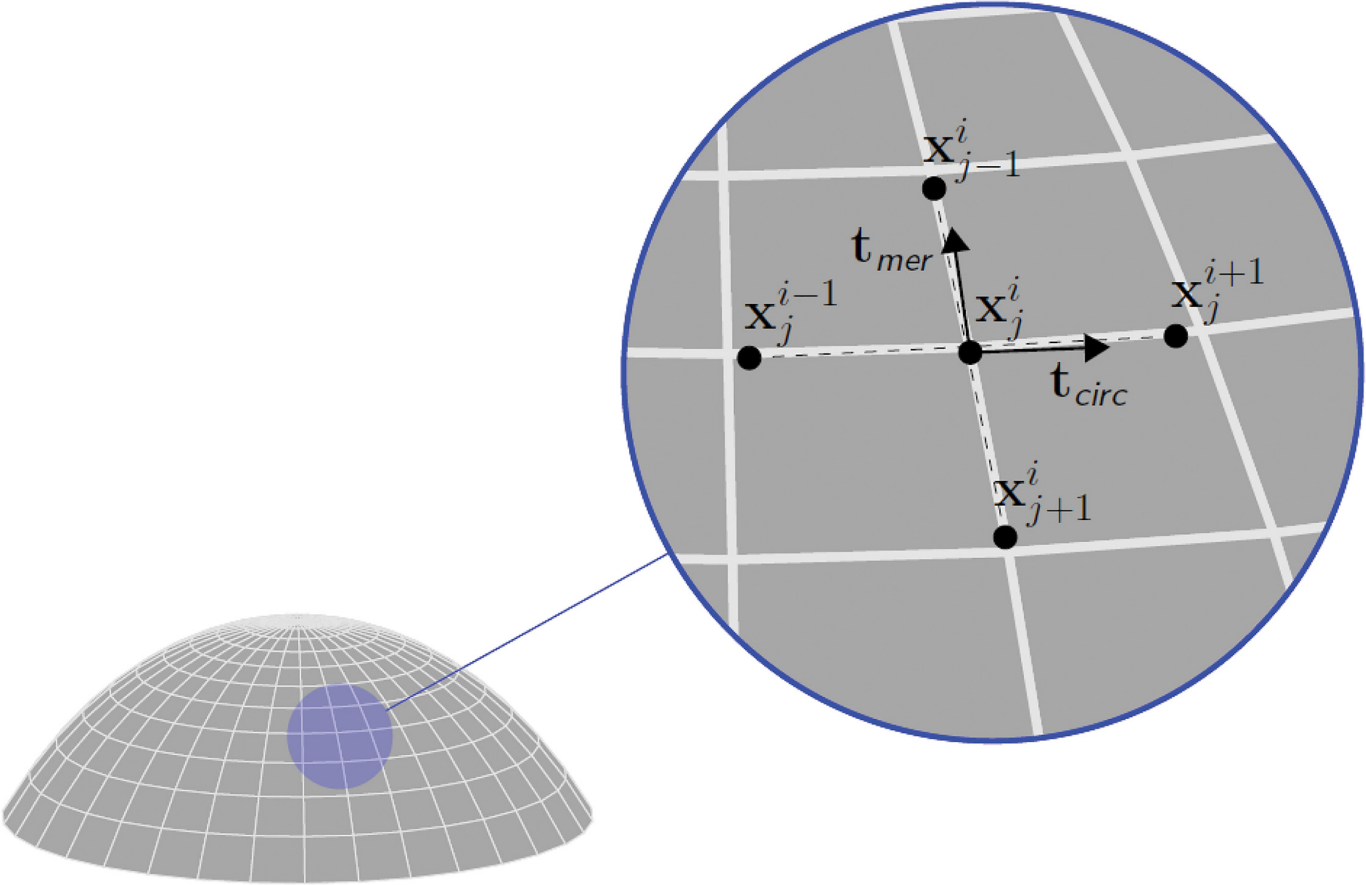
Orthogonal scheme used to compute the normal to the anterior corneal surface. The superscript index refers to meridians, the subscript index refers to circumferences. The circumferential tangent vector ***t***_circ_ is computed as the unit segment joining two adjacent points on the same circumference. The meridional tangent vector ***t***_mer_ is computed as the unit segment joining two adjacent points on the same meridian. The normal vector ***n***_*a*_ is obtained from the vector product between the two orthogonal unit vectors.

To construct the posterior surface, we rely on the thickness values measured by the topographer, without corrections for possible optical distortions. The thickness *t* of a shell is defined as the minimum distance between the anterior and posterior surfaces taken along the normal to the middle surface of the shell. For thin shells, the normal to the middle surface is very well approximated by the normal to the anterior surface. At each point ***x***_*a*_ of the discretized anterior surface, we compute the normal ***n***_*a*_ using the vector product between two segments ***t***_circ_ and ***t***_mer_, both tangent to the anterior surface, constructed from the points surrounding ***x***_*a*_ in the circumferential and meridian directions, respectively (see Fig 4)

$$\begin{array}{cc} t_{\text{circ}}=\frac{x_{j}^{i-1}-x_{j}^{i+1}}{\left|x_{j}^{i-1}-x_{j}^{i+1}\right|}, & t_{\text{mer}}=\frac{x_{j+1}^{i}-x_{j-1}^{i}}{\left|x_{j+1}^{i}-x_{j-1}^{i}\right|} \end{array},$$ (1)

where the superscript index *i* refers to the circumference and the subscript index *j* refers to the meridian. The inward normal to the anterior surface at the point derives as

$$n_{a}=\frac{t_{\text{mer}}\times t_{\text{circ}}}{\left|t_{\text{mer}}\times t_{\text{circ}}\right|}.$$ (2)

Then we set the point ***x***_*p*_, corresponding to ***x***_*a*_ on the posterior surface, along the normal at a distance equal to the thickness, as

$$x_{p}=x_{a}+t\;n_{a}.$$ (3)

In other words, the posterior corneal surface is obtained by moving along the normal the anterior surface points of an amount equivalent to the thickness.

**Fig 5:**
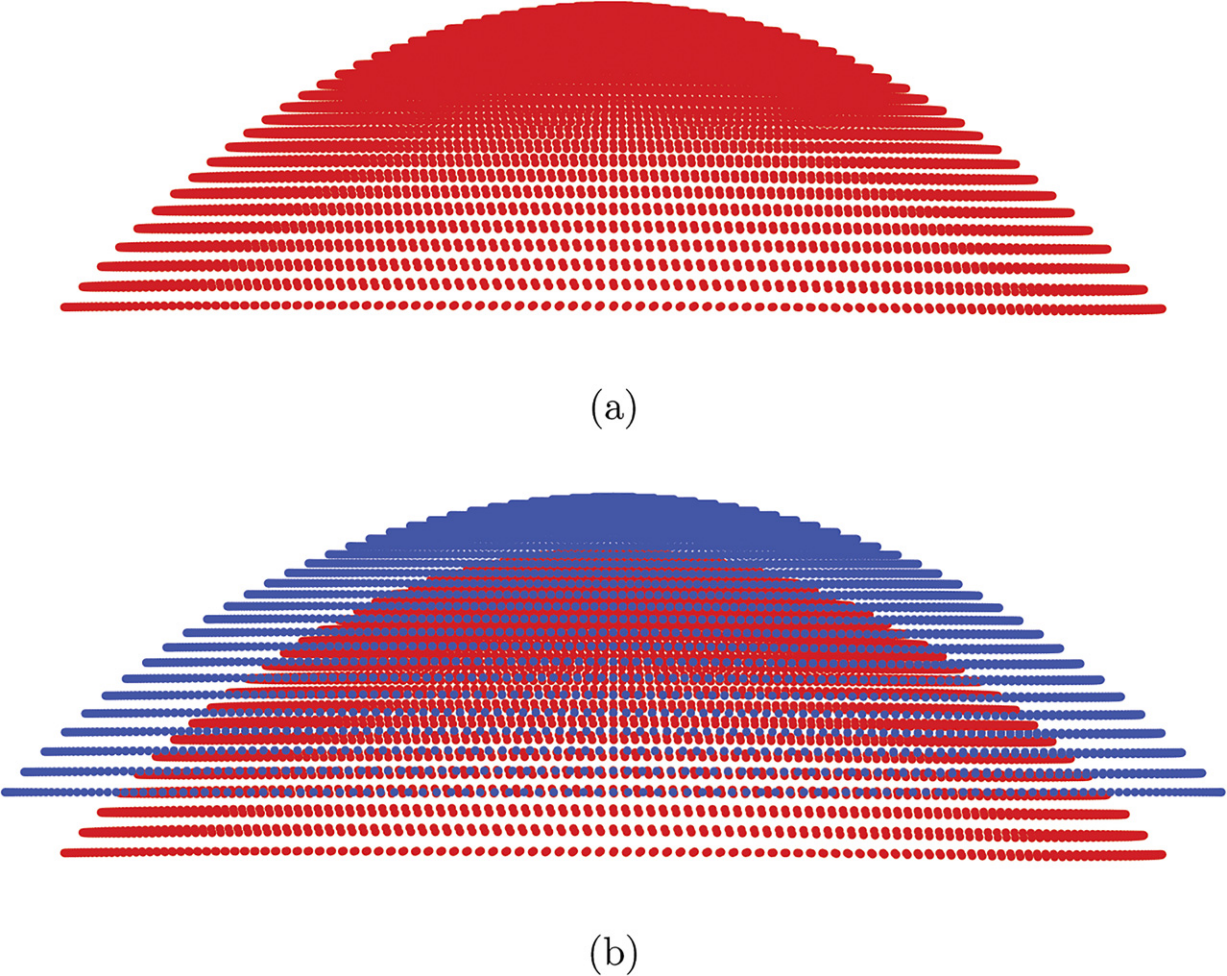
Cloud of points defining the posterior corneal surface. (a) Isolated posterior cornea surface points; (b) combined anterior and posterior corneal surfaces.

The procedure creates a posterior surface with a smaller curvature radius everywhere, and at the limbus the delimiting circumferential surface is cut orthogonal to the mean surface of the corneal shell, Fig 5. The resulting solid model of the cornea has a natural smooth shape, rather different from approximated solid models recently published [7].

#### Finite element discretization

**Fig 6:**
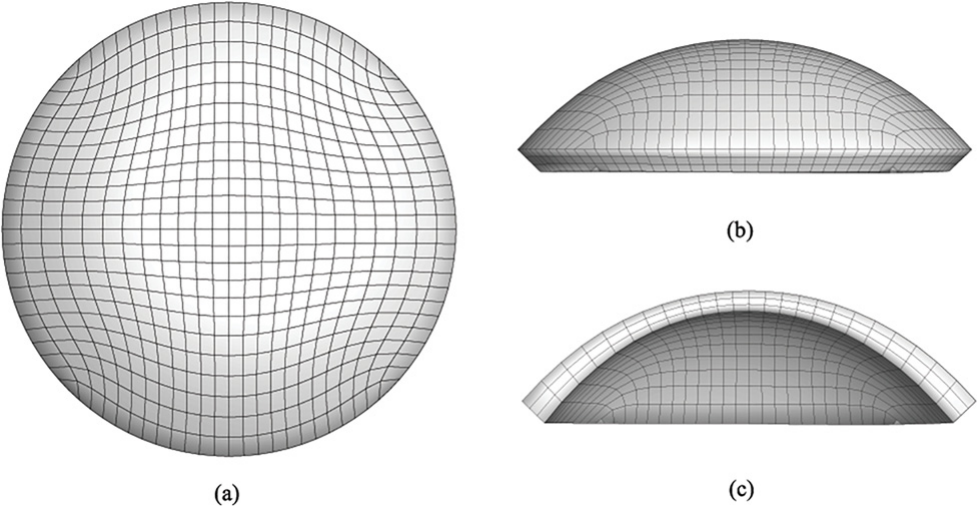
Finite element discretization of a patient-specific model of a human cornea in the physiological configuration. (a) anterior surface view; (b) NT side view; and (c) NT section. The boundary conditions for static analysis include constrained displacement of the limbus nodes in the radial direction, while the allowed displacements at the limbus mimic the free rotation of the corneal shell about the central limbus circumference.

An existing parametrized mesh generator for the cornea [17, 20] has been adapted to deal with the customized solid model of the cornea. By using a two-dimensional grid generator, the code creates a mesh discretized in 8-node brick elements with linear displacement interpolation. The discretization parameters are the number of elements along the principal diameter and the number of elements across the thickness. An example of patient-specific mesh is shown in Fig 6, and consists of 2500 nodes and 1728 brick elements, with three layers of elements across the thickness. The mesh generator uses a new algorithm based on Green functions [38] to interpolate the cloud of points with a continuous surface of equation

z=f(x,y). (4)

The quality of the interpolation has been assessed as follows. We computed the normalized distance between each node of the anterior surface of the mesh and the point of the cloud closest to it. In all the cases the sum of all the normalized distances was found < 1%. Thus the surface resulting from the interpolation algorithm reproduces with high fidelity the actual shape of the cornea, providing a customized geometrical model. Note that, in generating the geometrical model to be employed in numerical calculations, we must refer to the stress-free configuration of the cornea, i.e., the situation where the tissue is not loaded by the intraocular pressure (IOP). In-vivo measurements, though, describe the corneal geometry modified by the action of the IOP. The finite element code allows to recover the stress-free configuration through a simple iterative procedure, that has been explained in detail in [27] and will be briefly recalled in the next sections.

### Analytical model of the cornea’s geometry

Although the customized model of the cornea reconstructed from the topographer’s data is certainly the most proper for any numerical investigation, it is useful to characterize the geometry of the cornea with significant geometrical and refractive parameters, which can be easily obtained by interpolating the discrete surface with analytical models. In particular, a quick evaluation of the refractive power (RP) along a meridian can be obtained by the application of the thin lens equation,

$$\text{RP}=\frac{n_{c}-1}{R_{\text{ant}}}-\frac{n_{a}-n_{c}}{R_{\text{pos}}},$$ (5)

where *n*_*c*_ = 1.3375 and *n*_*a*_ = 1.336 are the refractive index of the cornea and the refractive index of the humor aqueous, respectively, and *R*_ant_ and *R*_pos_ are the curvatures of the anterior and posterior meridian of the cornea, respectively.

A good and useful approximation of the IOP pressurized anterior and posterior surfaces of the cornea is given by the biconic surface [20, 27, 39], whose sections are conic curves (ellipses, hyperboles, parabolas, and circumferences). Given a customized cornea, the optimal parameters of the best approximating biconic can be retrieved by using the least-square method. In cylindrical coordinates (*ρ*,*θ*) the biconic equation is given by

$$z\left(\rho ,\theta ;R_{s},R_{f},Q_{s},Q_{f},\theta_{s},z_{0}\right)=z_{0}-\frac{\rho^{2}A}{1+\sqrt{1-\rho^{2}B}},$$ (6)

Where

$$A=\frac{\cos^{2}\left(\theta -\theta_{s}\right)}{R_{s}}+\frac{\sin^{2}\left(\theta -\theta_{s}\right)}{R_{f}},$$ (7)

And

$$B=\left(Q_{s}+1\right)\;\frac{\cos^{2}\left(\theta -\theta_{s}\right)}{R_{s}^{2}}+\left(Q_{f}+1\right)\;\frac{\sin^{2}\left(\theta -\theta_{s}\right)}{R_{f}^{2}}.$$ (8)

In Eq (6), *R*_*s*_ and *R*_*f*_ define the maximum and minimum curvatures of the principal (steepest and flattest) meridians, *θ*_*s*_ defines the direction of steepest principal meridian with respect to the NT direction, *Q*_*s*_ and *Q*_*f*_ define the asphericity parameters in direction *θ*_*s*_ and $\theta_{s}+\frac{\pi }{2}$, respectively. The biconic surface reaches the maximum value *z*_0_ at *ρ* = 0. The principal conic sections can be either oblate (*Q*_*i*_ > 0) or prolate (−1 < *Q*_*i*_ < 0) ellipses, circumferences (*Q*_*i*_ = 0) or parabolas (*Q*_*i*_ = −1).

**Fig 7:**
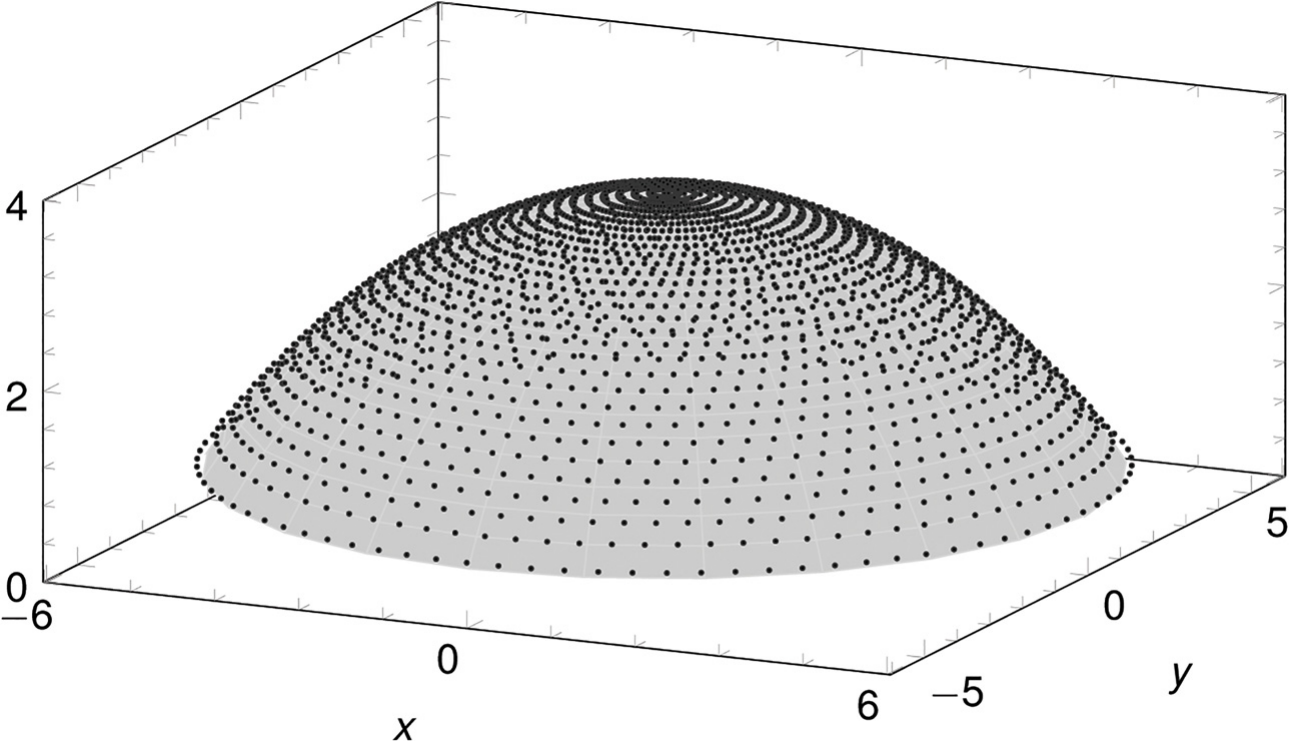
Comparison between the customized data points and the biconic surface for one of the patients considered in this study.

The optimal biconic parameters *R*_*s*_,*R*_*f*_,*Q*_*s*_,*Q*_*f*_,*θ*_*s*_,*z*_0_ of the anterior and posterior surfaces are computed by solving the following optimization problem:

$$\text{Err}=\min_{R_{s},R_{f},Q_{s},Q_{f,}\theta_{s},z_{0}}\;\sqrt{\overset{N}{\underset{i=1}{\sum }}{\left(z_{i}-z\left(\rho_{i},\theta_{i};R_{s},R_{f},Q_{s},Q_{f,}\theta_{s},z_{0}\right)\right)}^{2}},$$ (9)

where N is the number of the data points acquired from the topographer on the anterior (posterior) surface, *ρ*_*i*_,*θ*_*i*_,*z*_*i*_ are the cylindrical coordinates of the *i*-th data point. The optimization procedure provides an accurate analytical model of the customized surface. The comparison between the approximated biconic surface and the solid model obtained by spline interpolation of the topographer data shows that the two surfaces cannot be distinguished, Fig 7.

The mean final error of problem (9) for all the corneas considered in this study is 2.42±1.1%. The obtained parameters can be used to evaluate average refractive properties of the examined corneas.

The least square method is an optimization technique based on the Newton-Raphson algorithm, and it can only find local minima. Although theoretically other optimal sets of parameters may exist, the regularity of the loading conditions for the physiological cornea rules out the possibility to converge towards unrealistic sets of parameters.

### Material model for the stroma

According to our previous approaches, and considering the structural relevance of the stromal layer with respect to external membranes, we assume that the thinner layers of the cornea can be disregarded and assign to them the same material properties of the stroma. The main motivation for this simplification is the lack of information about the mechanical properties of the external layers [27]. The mechanical behavior of the stroma is described through an anisotropic hyperelastic material model characterized by a strain energy density function *ψ* decomposed additively into three independent terms:

$$\psi =\psi_{\text{vol}}\left(J\right)+\psi_{\text{iso}}\left({\bar{I}}_{1},{\bar{I}}_{2}\right)+\psi_{\text{aniso}}\left({\bar{I}}_{4,1}^{*},{\bar{I}}_{4,2}^{*}\right).$$ (10)

**Fig 8:**
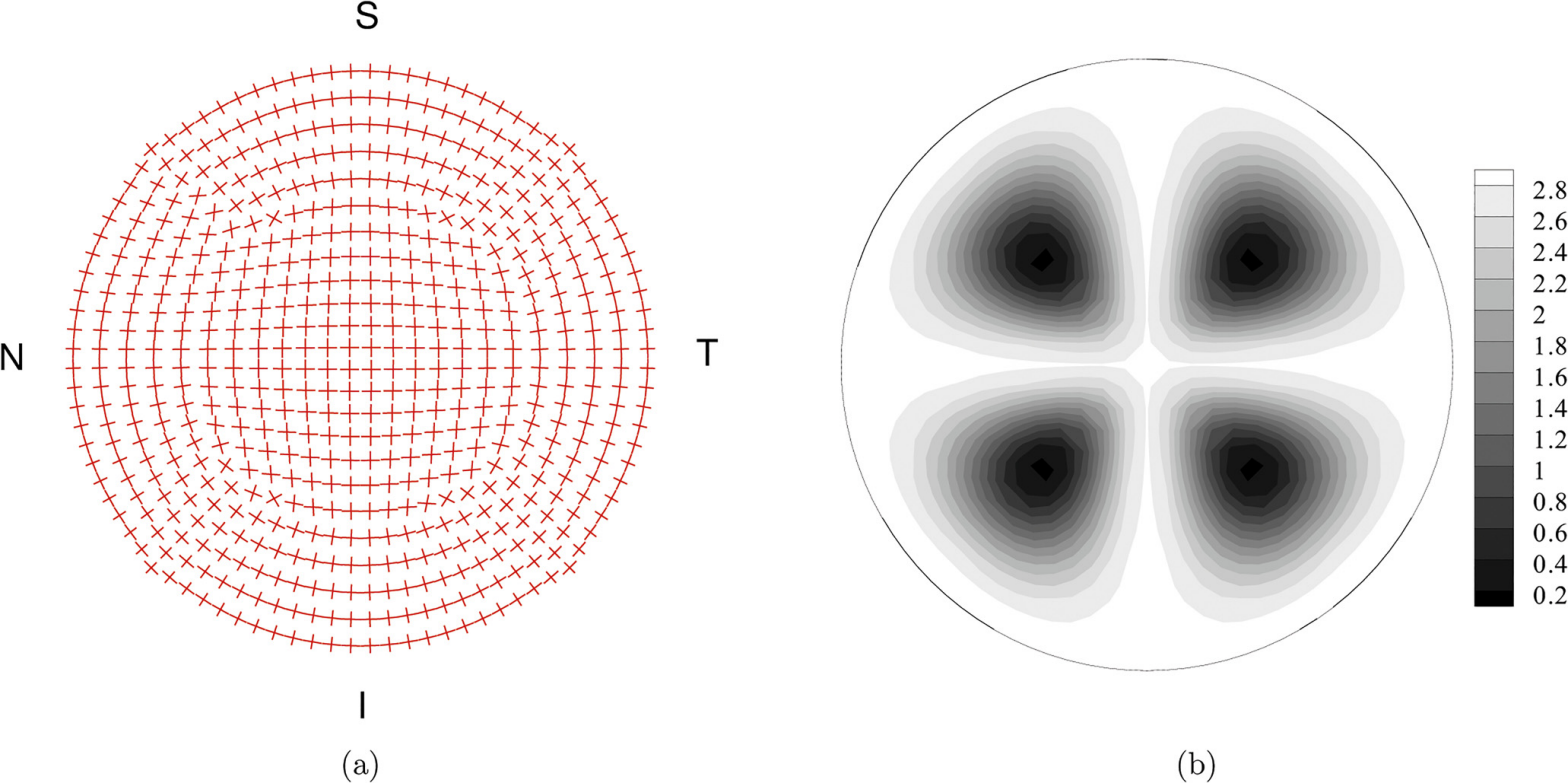
Fiber organization in the top layer of the finite element discretization of the cornea model. (a) Mean orientation of the two sets of fibers. (b) Map of the von Mises coefficient *b* for the statistical distribution of the orientation. High values of *b* define highly oriented set of fibers. Low values of *b* define nearly isotropic orientations.

The first term, *ψ*_vol_, accounts for volume changes, and it is assumed to be dependent on the determinant of the deformation gradient **F**, or jacobian *J* = det **F**. For incompressible materials, this term must be considered as a penalty contribution to contrast undesired changes of the volume. The expression of *ψ*_vol_ is:

$$\psi_{\text{vol}}\left(J\right)=\frac{1}{4}K\left(J^{2}-1-2\log J\right),$$ (11)

where *K* is the bulk modulus. The term *ψ*_iso_ describes the behaviour of the isotropic aspects of the material behaviour, including the underlying matrix and the portion of randomly distributed fibrous reinforcement. As usual, *ψ*_iso_ is assumed to be dependent on the first and second invariants of the modified Cauchy-Green deformation tensor $\bar{C}={\bar{F}}^{T}\bar{F}$ where F¯=J−13F. We select an expression corresponding to the Mooney-Rivlin model

$$\psi_{\text{iso}}\left({\bar{I}}_{1},{\bar{I}}_{2}\right)=\frac{1}{2}\mu_{1}\left({\bar{I}}_{1}-3\right)+\frac{1}{2}\mu_{2}\left({\bar{I}}_{2}-3\right),$$ (12)

with *μ* = *μ*_1_ + *μ*_2_ the shear modulus. The third term *ψ*_aniso_ describes the anisotropic behavior, including the effects of the microstructure of the fibrils. It usually depends on the modified tensor $\bar{C}$ and on particular vectors or tensors describing the intrinsic structure of the material. In the present calculations we refer to the particular model described in [37], of the form

$$\psi_{\text{aniso}}\left({\bar{I}}_{4,1}^{*},{\bar{I}}_{4,2}^{*}\right)=\overset{2}{\underset{F=1}{\sum }}\frac{1}{2}\frac{k_{1F}}{k_{2F}}\exp\left[k_{2F}{\left({\bar{I}}_{4,F}^{*}-1\right)}^{2}\right]\left(1+K_{F}^{*}\sigma_{I_{4,F}}^{2}\right),$$ (13)

where *k*_1*F*_ are the stiffness parameters and *k*_2*F*_ are dimensionless rigidity parameters. Overall, the material model for the cornea needs the assignment of seven material parameters (*K*,*μ*_1_,*μ*_2_,*k*_11_,*k*_12_,*k*_21_,*k*_22_). The material model requires also the definition of the spatial distribution of the main orientation ***a***_*F*_ of two sets of fibrils and of the concentration parameter *b*_*F*_(*ρ*,*θ*,*z*), that describes the spatial dispersion of the fibrils about the main orientation. It is now well known that the organization of the fibrils in the cornea follows a particular pattern with dominant orientation in the nasal-temporal (NT) and superior-inferior (SI) directions [40, 41]. The variability of the interlacing and of the dispersion of the fibrils orientation across the thickness has been recently elucidated [35, 36]. According to recent findings, the organization of the collagen fibrils in the deep cornea is better modeled by a planar distribution of the fibrils orientation than a fully tridimensional distribution. In the present calculations, though, we kept using the model already employed and validated in previous studies, since here we are interested in the geometrical aspects of the corneal modeling, while the investigation on the material model will be object of an on-going study. In the outermost layer of the cornea model, fibrils are organized according to the model described in [17, 34]. In the central part of the model, fibrils follow the NT and SI orientation, and progressively rotate the orientation while moving towards the limbus, where the main set of fibrils runs circumferentially. A secondary, more compliant, set of fibrils runs in the radial direction to guarantee the correct mechanical behavior of the shell, Fig 8A. Everywhere, the spatial orientation of collagen fibrils follows a transversely isotropic and *π*-periodic, normalized, von Mises distribution:

$$\begin{array}{cc} \rho \left(\Theta \right)=\frac{1}{2\pi \;I}\exp\left(b\cos2\Theta \right), & I=\frac{1}{\pi } \end{array}\overset{\pi }{\underset{0}{\int }}\exp\left(b\cos2\Theta \right)d\mathrm{\Theta ,}$$ (14)

with a concentration parameter *b* distributed according to Fig 8B.

### Finite element model

In the numerical calculations we model only the cornea and not the whole eye. The reason for this choice is that, with the current advanced diagnostic instruments used in ophthalmology, it is impossible to identify the exact geometry, the correct material model, and the true material properties of all the parts of the eye. It is well known, though, that the deformed shape of the cornea is considerably affected by displacements at the limbus [42]. To account for the missing parts of the eye, in our model the displacement boundary conditions are chosen so as to describe at best the support offered by the limbus. The cornea is joined with the sclera, which works as a compliant support allowing displacements and rotations. Displacements in the radial direction are of scarce relevance, for the observed stiffness of the limbus (less thick that the one of the central cornea). Also displacements in the z direction cannot be consider, since they do not modify the shape of the cornea. The most important displacement at the boundary is therefore the rotation about the limbus mean circumference, which is included in our analysis [20, 27, 34].

The loading conditions considered here include an increasing IOP from 0 mmHg to 40 mmHg; the reference physiological IOP is 18 mmHg. We consider geometries related to preoperative and postoperative corneas. At each stage of the loading under a growing IOP, the numerical code computes the best fitting biconic surface, and the corresponding parameters are used to estimate the refractive power in the direction of NT and SI meridians.

The stress-free geometry and the material parameters of the model are unknown, and they have to be identified using an inverse analysis procedure that will be briefly recalled in the next paragraphs.

#### Stress-free configuration identification

As already said, the solid model reconstructed from the topographer data corresponds to the stressed configuration of the cornea, which reacts to the individual IOP. Before performing a stress analysis, the geometry of the solid model should be pushed back to the natural configuration, corresponding to a null IOP and to a null stress state. The finite element code used in the present study is equipped with an automatic recovery (or identification) of the unstressed configuration of the cornea. The identification procedure works iteratively by comparing the coordinates of the anterior and posterior surfaces of the cornea as provided by the clinical measurements and the ones obtained from the stress analysis of the finite element model under the physiological IOP. The data ***x*** obtained from clinical measurements are used to build a target finite element mesh *M*_0_, with the (stressed) coordinates expressed as:

$$x=X_{k}+u_{k},$$ (15)

where ***X***_*k*_ are the unknown stress-free coordinates at the iteration *k* and ***u***_*k*_ are the computed nodal displacements at the iteration *k*. The procedure begins by setting ***X***_0_ = ***x***. Then the code performs a static analysis by applying the physiological IOP on the posterior surface of the cornea. At the end of the *k*-th iteration, the stress-free coordinates are recomputed as

$$X_{k+1}=x-u_{k}.$$ (16)

The procedure continues until the norm of the difference in coordinates between two iterations becomes smaller than a specified tolerance

$$\left|X_{k+1}-X_{k}\right|\leq \varepsilon .$$ (17)

**Fig 9:**
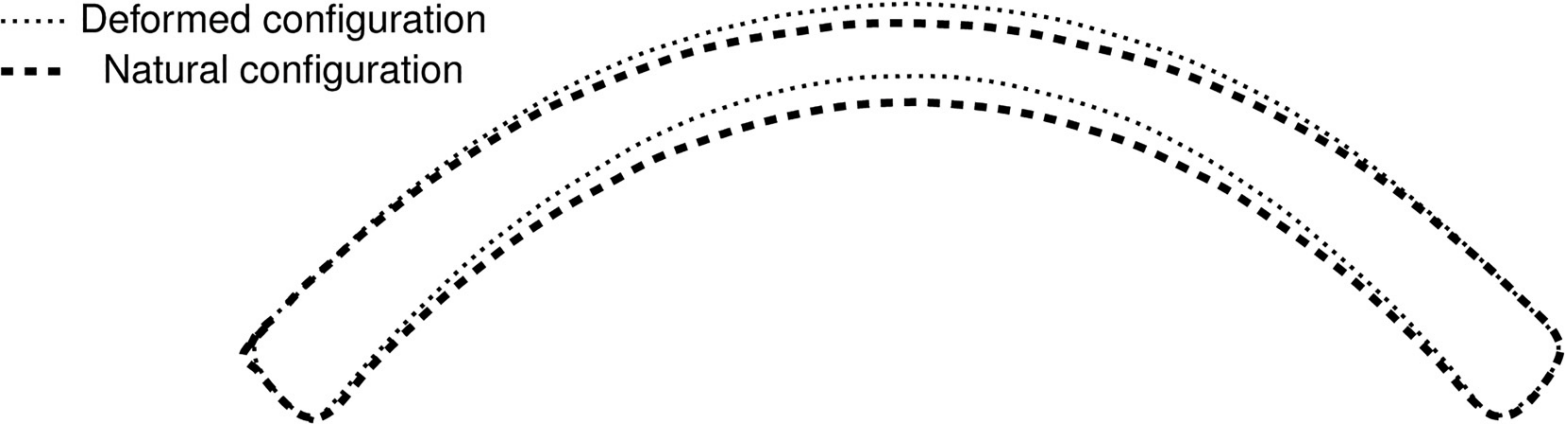
NT meridian section: comparison between the stressed (or deformed) configuration and the stress-free (or natural) configuration for the patient IV.

In the present case it has been taken *ε* = 10^−6^. A comparison between the natural and the deformed configuration for one of the patients considered in the present study is shown in Fig 9.

#### Material parameter identification

Once the material model has been selected and the intrinsic structure of the collagen fibrils has been assigned, the identification of the material parameters cannot be disjointed from the identification of the stress-free configuration, since the two aspects (geometry and mechanical response) are intrinsically connected. Following the procedure described in [27], in the present calculations the material properties for the different patients are estimated by alternating the simulation of the preoperative and postoperative conditions, and determining for each condition the stress-free configuration.

We assume that, in the stress-free configurations, the geometry of posterior surface of the cornea must be equal for preoperative and postoperative cornea. This assumption is supported by the observation that no permanent corneal damage was observed in the eyes after the surgical procedure, suggesting that the stromal material properties do not undergo changes after the tissue ablation. Since the surgery modifies the thickness of the cornea, the structural properties change and also the mechanical response of the cornea changes. The identification procedure relies on the comparison between two different structural conditions for the same material. The identification of the material parameters is achieved with an automated procedure that varies the material properties according to the differences observed in the geometry of the stress-free posterior surfaces of the preoperative and postoperative cornea. Stiff (soft) material parameters result in a postoperative posterior surface with a smaller (larger) radius than the preoperative. We note that in the material property identification procedure we assume the invariance of the “stress free” or “natural” configuration of the posterior surface of the cornea, while the physiological configuration will be necessarily modified. By definition, the natural configuration is exempt by any deformation process (elastic, viscous, hydric, electric, and others). The assumption taken might be rather strong, but it is fully consistent and coherent with the choice of a purely elastic, damage free, dry, material model. Whenever a more sophisticated material model accounting for material damage of swelling processes will be considered, this procedure needs to be reconsidered and modified accordingly.

The solution of this procedure is not unique, it depends on how many parameters are allowed to vary. Moreover, the results are strongly dependent on the chosen material model.

We began by calibrating the material properties for one of the eyes of a patient. Next, we tried to use the same set of material properties to identify the stress free configuration for other patients. The procedure yielded preoperative and postoperative geometries characterized by very similar posterior surfaces: in all cases, the total normalized distance between the posterior surface nodes of the two configurations was found < 1%. Thus we used the same set of material parameters, listed in Table 1, in all the subsequent calculations.

**Table 1:** Material parameters adopted in the numerical calculations for the five patients.

*K*(MPa)	*μ*_1_(MPa)	*μ*_2_(MPa)	*k*_11_(MPa)	*k*_21_	*k*_12_(MPa)	*k*_22_
5.5	0.06	-0.01	0.04	200	0.04	200

## Results

In this study we considered five patients treated with PRK for myopia (Patients I, II, III), astigmatism (Patient IV) and myopic astigmatism (Patient V). Preoperative and postoperative clinical refractive data (spherical power S, cylindrical power C, and angle *α*) for the five patients are collected in Table 2. The effect of the laser ablation on the cornea can be analyzed by considering the variation under growing IOP in the apical displacement, in the geometrical parameters and RP, and in the stress distribution.

**Table 2:** Preoperative and postoperative clinical refractive data for the five patients.

Patient	Preoperative			Postoperative		
	S (D)	C (D)	*α* (deg)	S (D)	C (D)	*α* (deg)
I	38.3	-0.2	23°	34.2	-0.3	25.5°
II	41.2	-0.2	161°	35.0	-0.7	171°
III	41.3	-0.7	154.5°	33.7	-0.9	160.5°
IV	43.6	-1.9	145°	39.1	-0.4	12°
V	42.9	-1.8	34.5°	40.8	-0.5	11°

### Apical displacement

**Fig 10:**
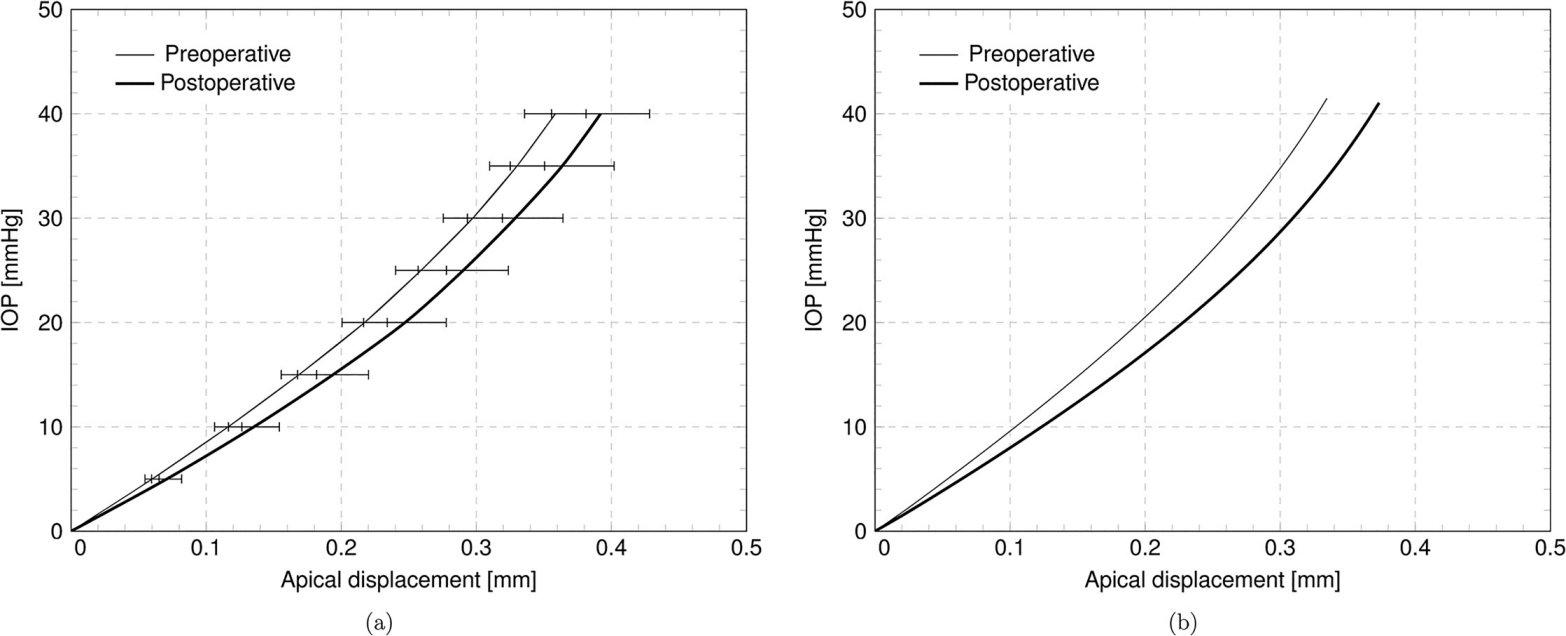
Numerical results. **IOP versus apical displacement curves, in the preoperative and postoperative case.** (a) Average over five patients. (b) Results for patient IV.

A significant synthesis of the experimental or numerical mechanical response of the cornea is given by the plot of the IOP versus the displacement of the cornea’s apex in the direction of eye axis. Fig 10A shows the average IOP-apical displacement curve for the five eyes considered in this study, in the preoperative and postoperative configurations. The average preoperative and postoperative maximum apical displacements are 0.3645 ± 0.023 mm and 0.3960 ± 0.036 mm, respectively. The postoperative average displacement has 8% increment with respect to the preoperative case. Table 3 collects the numerically computed apical displacement at physiological IOP for the five patients, including the relative difference. The individual effect of the ablation on a single patient is visualized in Fig 10B, showing the plot of the IOP versus apical displacement for Patient IV.

**Table 3:** Numerically computed corneal apical displacement in the preoperative and postoperative configurations for the five patients. Absolute values and relative increment.

Patient	Disease	Preoperative (mm)	Postoperative (mm)	Increment %
I	Myopia	0.2146	0.2585	20%
II	Myopia	0.2132	0.2233	5%
III	Myopia	0.1986	0.2547	28%
IV	Astigmatism	0.1784	0.2086	17%
V	Myopia and Astigmatism	0.1869	0.1935	4%

### Geometrical parameters and refractive power

**Fig 11:**
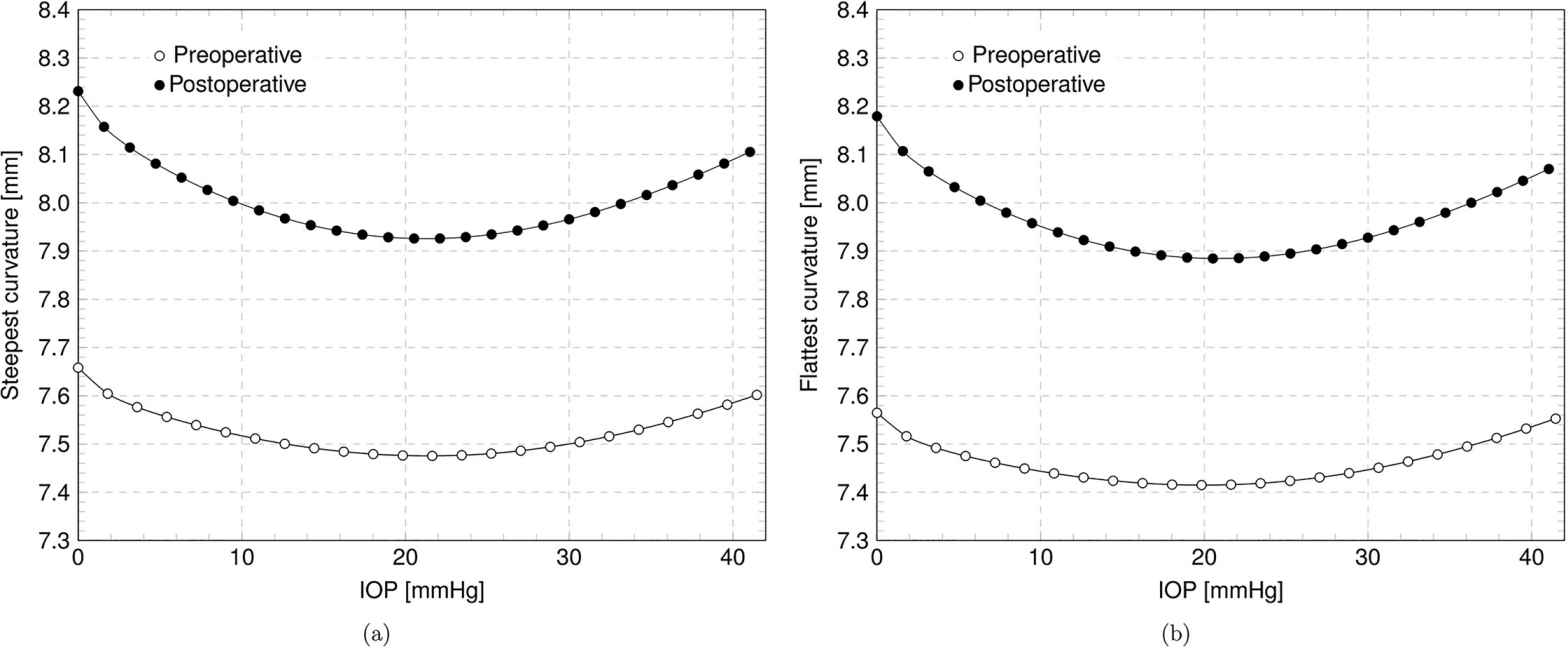
Numerical results for the patient IV in the preoperative and postoperative case. (a) Steepest curvature versus IOP. (b) Flattest curvature versus IOP.

**Fig 12:**
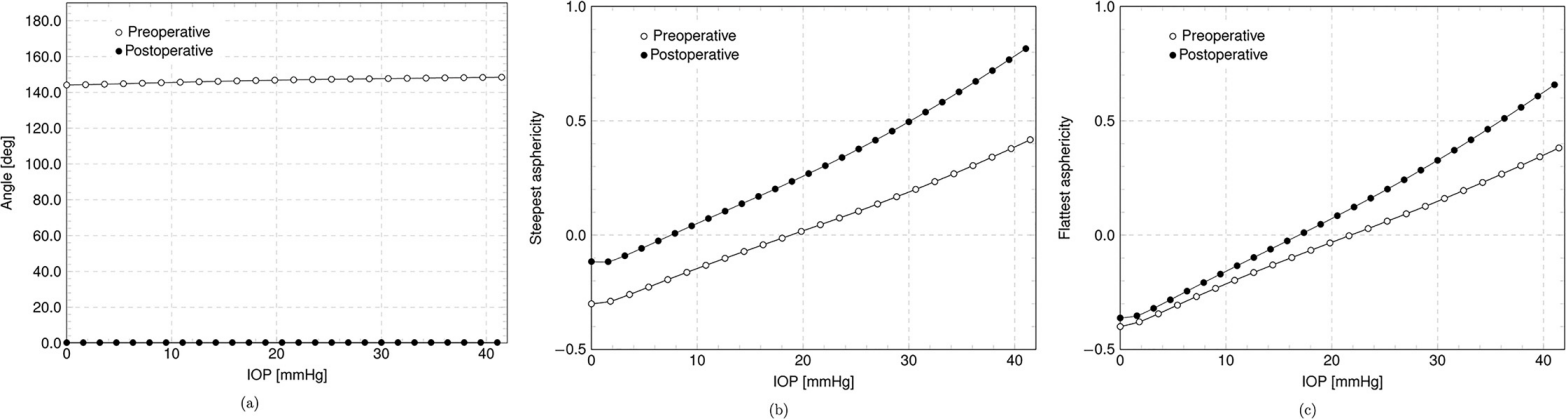
Numerical results for the patient IV in the preoperative and postoperative case. (a) Steepest meridian and NT direction angle versus IOP. (b) Steepest meridian asphericity coefficient versus IOP. (c) Flattest meridian asphericity coefficient versus IOP.

**Fig 13:**
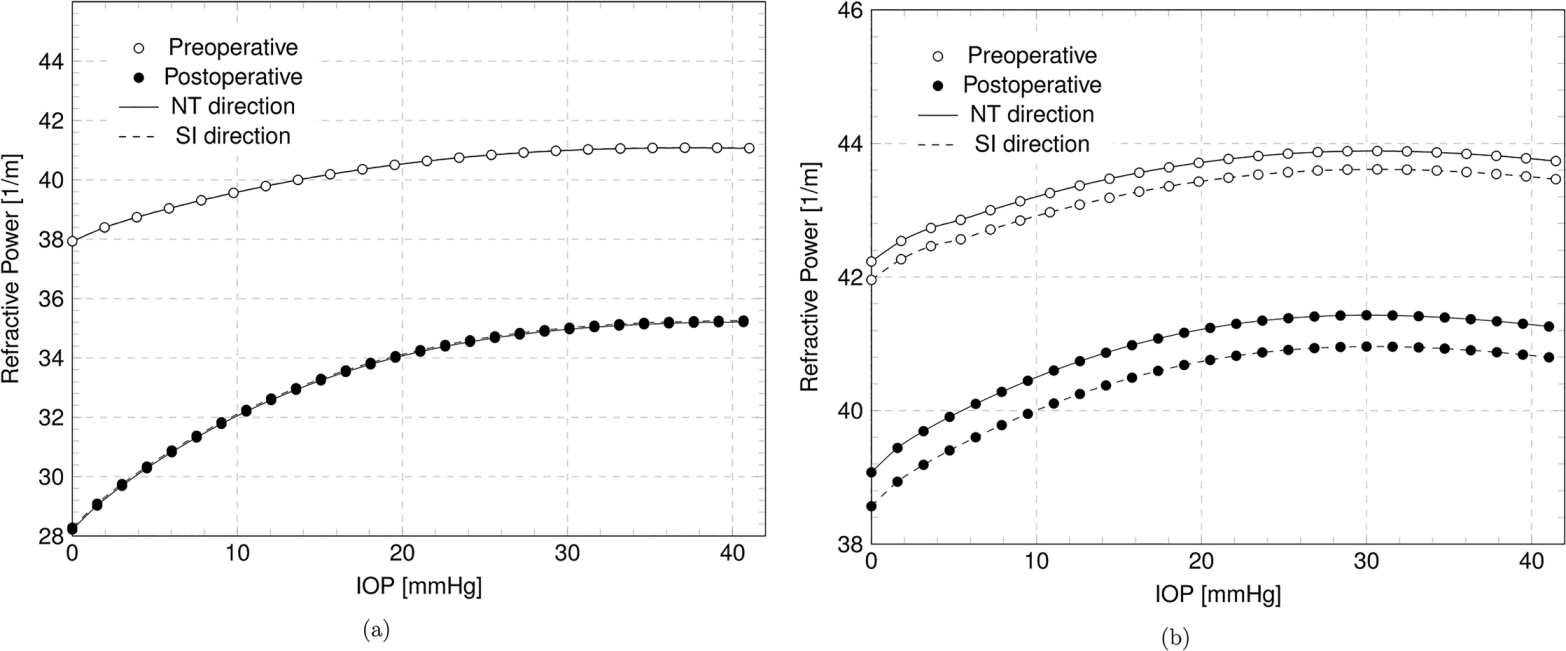
Numerical results in the preoperative and postoperative case. Refractive power in the NT and SI direction versus IOP. (a) Patient III, myopic. (b) Patient IV, astigmatic.

The reprofiling of the cornea due to laser ablation modifies the geometry inducing a change in the best fitting biconic parameters. The variation of the biconic parameters with IOP are visualized in the Figs 11–12. Fig 11 shows, for Patient IV, the steepest and the flattest meridian curvatures versus the IOP for the preoperative and postoperative corneas, while Fig 12 shows the angle and the asphericity coefficients in the steepest and flattest meridian directions versus the IOP. Fig 13 shows the variation of the RP in the NT and SI direction with IOP in preoperative and postoperative cases for Patient III, treated for myopia, and Patient IV, treated for astigmatism.

### Stresses

**Fig 14:**
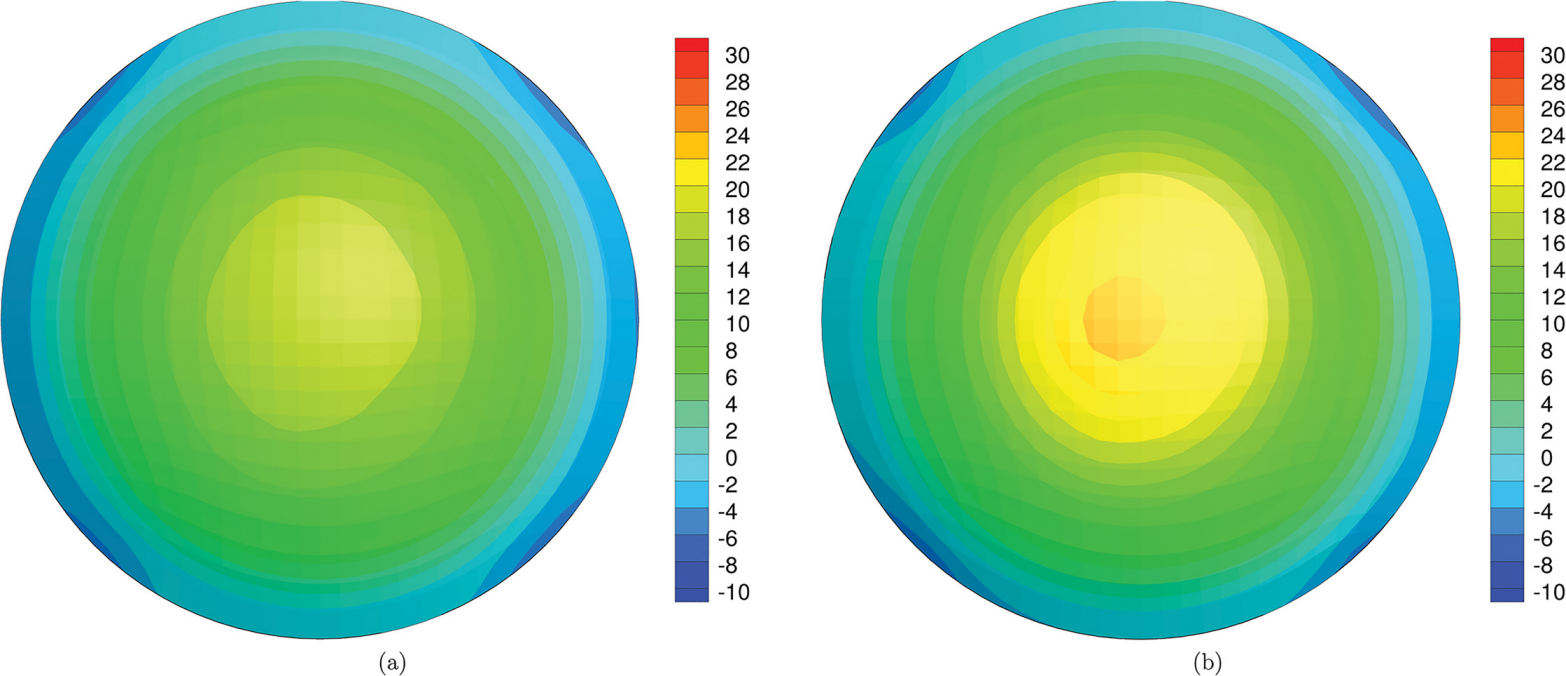
Numerical results for the patient III, treated with the deepest ablation. Contour levels of the NT component of the Cauchy stress (kPa) on the anterior surface of the cornea, at IOP 18 mmHg. (a) Preoperative condition. (b) Postoperative condition.

**Fig 15:**
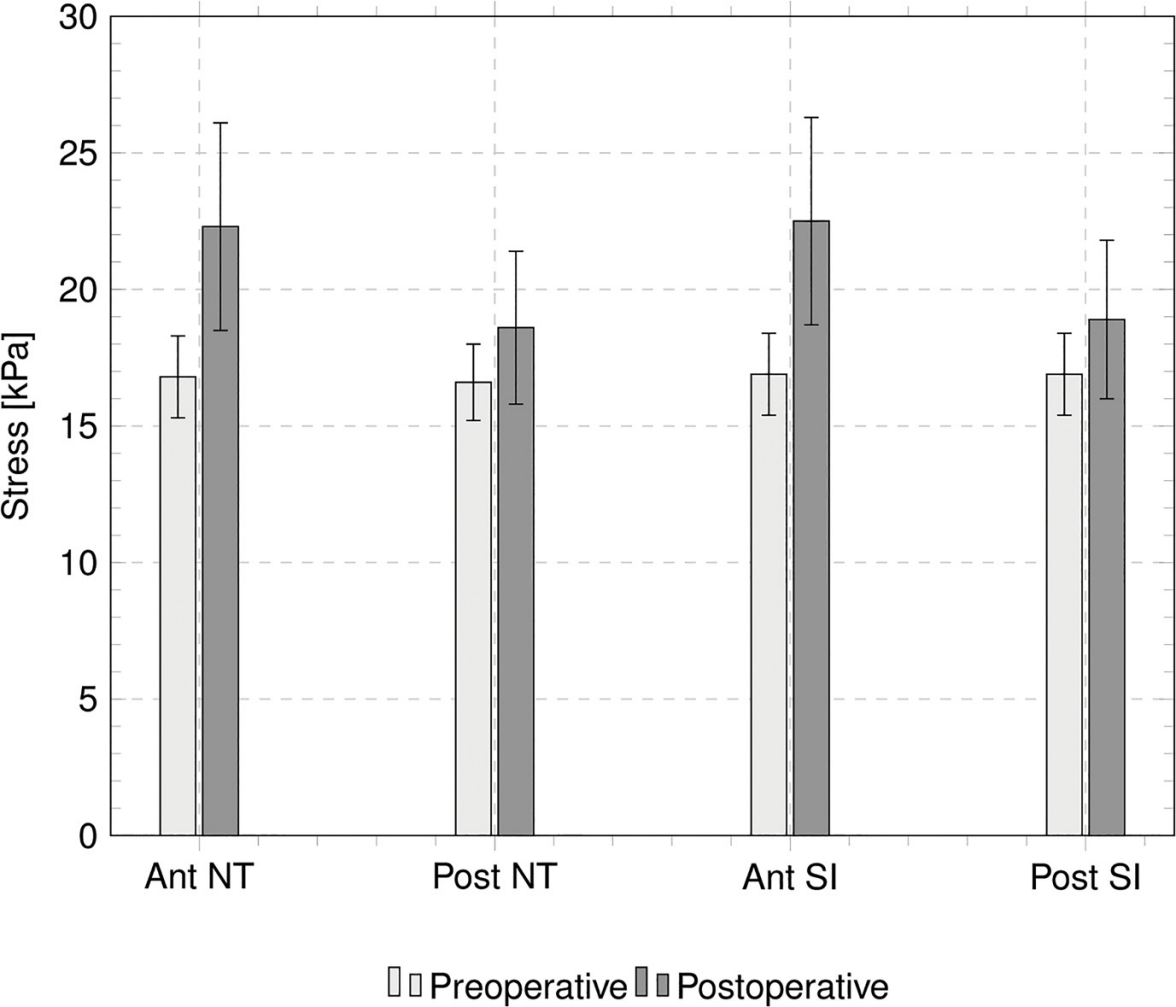
Numerically evaluated Cauchy stress components, averaged over five patients, at the center of the anterior and posterior surfaces of the cornea. Horizontal axis labels refer to: (1) NT stress component on the anterior surface, (2) NT stress component on the posterior surface, (3) SI stress component on the anterior surface, (4) SI stress component on the posterior surface. White and black bars denote the preoperative and postoperative stress, respectively.

**Fig 16:**
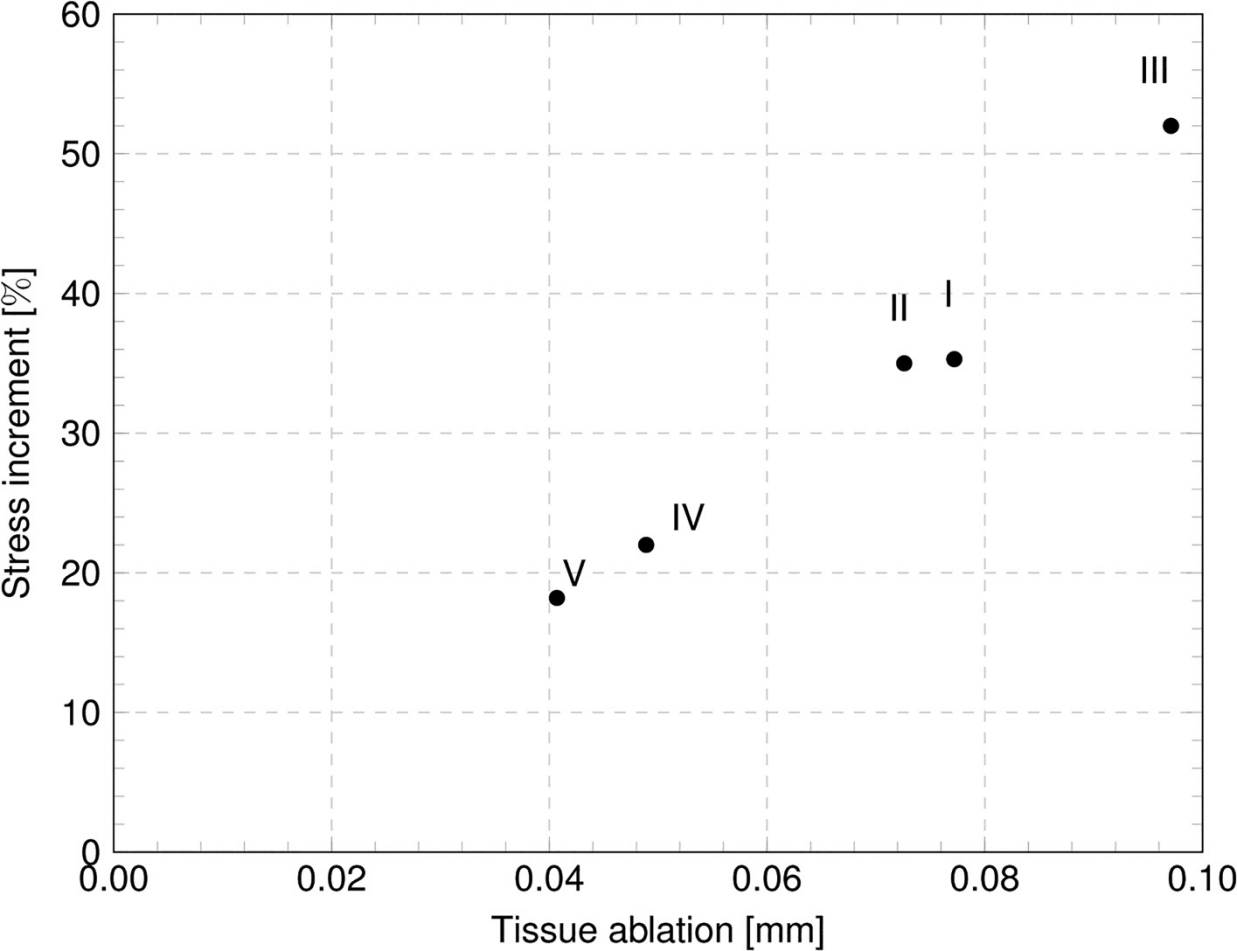
Relative postoperative increment of the stress in the NT direction, at the apex of the anterior surface of the cornea, as a function of the ablation depth.

Static analyses provide the stress distributions with varying IOP, for the preoperative and the postoperative cases. Fig 14 shows the stress distribution for Patient III, treated with the deepest ablation, at 18 mmHg IOP; contour levels refer to the NT component of the Cauchy stress. For the five patients, Tables 4 and 5 report the values of the NT and SI Cauchy stress component at the apex of the anterior surface of the cornea, at 18 mmHg IOP, and compare the stress in the preoperative and postoperative conditions, in terms of absolute values and relative increment. Fig 15 compares the NT and SI Cauchy stress at the cornea’s apex, averaged over the five patients, at 18 mmHg IOP, computed in proximity of the anterior and posterior surfaces, for both the preoperative and postoperative conditions. The plot clearly shows the increment of the average stress in the postoperative cases. The same average stresses, including the standard deviations, are reported in Table 6. The average increment of the stress after surgery is 11% for the posterior surface and 33% for the anterior surface. For the five patients, Fig 16 shows the relative postoperative increment of the Cauchy stress as a function of the average ablation depth. The average ablation depth has been computed by comparing the preoperative and the postoperative data on the corneal thickness in the central 3 mm radius (optical zone), as provided by the topographer measurements. The stress values reported in Fig 16 refer to the NT component, computed on the anterior surface, at 18 mmHg IOP.

**Table 4:** Values of the Cauchy stress (in kPa) at the corneal apex, at the physiological IOP 18 mmHg, in the NT direction, anterior surface.

Patient	Disease	Preoperative (kPa)	Postoperative (kPa)	Increment %
I	Myopia	18.8	25.5	35.3%
II	Myopia	16.8	22.5	35.0%
III	Myopia	17.6	26.6	52.0%
IV	Astigmatism	15.1	18.4	22.0%
V	Myopia and Astigmatism	15.7	18.6	18.2%

**Table 5:** Values of the Cauchy stress (in kPa) at the corneal apex, at the physiological IOP 18 mmHg, in the SI direction, anterior surface.

Patient	Disease	Preoperative (kPa)	Postoperative (kPa)	Increment %
I	Myopia	19.0	25.8	35.5%
II	Myopia	16.8	22.6	34.6%
III	Myopia	17.6	26.8	52.5%
IV	Astigmatism	15.3	18.6	21.5%
V	Myopia and Astigmatism	15.9	18.8	18.8%

**Table 6:** Average values of the Cauchy stress (in kPa) at the corneal apex, at the physiological IOP 18 mmHg.

	Preoperative (kPa)	Postoperative (kPa)	Increment %
Anterior NT	16.8 ± 1.5	22.3 ± 3.8	33.1%
Anterior SI	16.9 ± 1.5	22.5 ± 3.8	33.2%
Posterior NT	16.6 ± 1.4	18.6 ± 2.8	11.5%
Posterior SI	16.9 ± 1.5	18.9 ± 2.9	11.9%

## Discussion

Laser refractive surgery modifies, through ablation of the stromal tissue, the curvature of the anterior surface of the cornea, inducing changes in the geometry, including the elevation of the apex. Geometry changes are direct, due to the tissue ablation, and indirect, due to the modified mechanical response of a shell, which has a thinner thickness but is subject to the same IOP.

The aim of laser corneal refractive surgery is to modify selectively the local curvature of the cornea to obtain along any meridian the desired refractive power. The consequence of the ablation is the modification of the global parameters that govern the mathematical description of the corneal shape, including the overall important apical displacement.

In the present study, we used an improved numerical model of the human cornea, described in [17, 20, 27], to analyze patient-specific geometries of five patients undergoing corneal refractive surgery. The focus of this research is the construction of accurate solid models from geometrical data obtained from corneal topographers, therefore the aspects concerning the material model have been left aside. Corneal topographers are equipped with software able to extract geometrical data from images and pachimetry measurements. A specific code has been developed to complete the geometrical data, often affected by gaps, and to construct a finite element model of the cornea reacting to the action of the physiological IOP. The code is also able to retrieve the stress-free (corresponding to zero IOP) configuration of the cornea, which is necessary to evaluate the actual stress state. The model has been used to perform quasi-static analyses of the corneas of five patients, which underwent laser refractive surgery to treat myopia or astigmatism. The analysis involved the preoperative and the postoperative corneas, and provided a wealth of numerical results, in terms of displacements, strain and stresses.

As scalar parameter, the apical displacement is particularly convenient to be analyzed statistically, and correlations with other measurements can be established. Although the apical displacement alone is insufficient to assess the predictability and the reliability of a numerical model, when combined with the two curvatures along SI and NT it can describe adequately the numerical results of patient-specific simulations. In the present study, the apical displacement is an unknown variable which is included in the diagnostic measurements of the cornea through the topographer. The plots in Fig 10 are outcomes of numerical simulations that cannot be directly compared to experimental data. However, given the accurate geometrical description of the cornea shape, the plots are realistic and show the typical stiffening IOP versus apical displacement curve that can be found in several experimental studies [13, 43, 44]. By comparing the mechanical response of preoperative and postoperative corneas, it is clearly shown that the thinning due to the ablation reduces the stiffness of the cornea, which shows a more compliant behavior, or a higher sensitivity to the IOP.

The analyses conducted in this study are able to provide the whole displacement field of the corneal surfaces as a function of the loading parameter, i.e., the IOP. The deformability of the structure can be monitored through the analysis of the parameters that describe at best the shape of the cornea within the optical zone, i.e., the biconic function. Figs 11–12 show the variability of the geometrical parameters with the IOP in the preoperative and postoperative cases. When undergoing a treatment for myopia or astigmatism, the curvature radius of the steepest and flattest meridian is increased, see Fig 11 (patient IV). However, the curvature changes with the IOP and the minimum radius is reached in proximity of the physiological IOP. IOPs outside the physiological range cause an increase of the radii (i.e., a flattening of the corneal surface) and a consequent reduction of the corneal visual acuity.

Fig 12 shows the variation of other biconic parameters with IOP and compares the preoperative and postoperative values for patient IV. It is interesting to observe how the angle *θ*_*s*_ between the NT axis and the steepest meridian has been fully corrected during the intervention, leading to a zero angle in the postoperative case, Fig 12A. In general, asphericity parameters are not specifically addressed in refractive surgery and the resulting values are more a consequence of the variation of other major parameters such as curvature and ablation depth. In the particular case here considered, the asphericity parameters for the patient IV show a postoperative increase, but maintain the same sensitivity to IOP observed in the preoperative conditions.

According to the thin lens Eq (5), the anterior and posterior curvatures of a meridian are strictly related to the RP. With reference to the myopic Patient III, Fig 13A shows that the RP, independent of the meridian, is rather stable in the range of the physiological IOP, but the sensitivity to low IOPs increases in the postoperative case. Another observation arising from the RP plot is that the maximum RP in a postoperative cornea is generally reached at a lower IOP with respect to the preoperative cornea. Fig 13B shows the variation of the RP with IOP for the astigmatic Patient IV. The RP has been reduced by flattening both the NT and the SI meridian, but the preoperative astigmatism has been preserved. In this case the ablation has not shifted the maximum RP to lower IOP. Fig 13 shows a 2 D variation of the corneal refractive power due to 10 mmHg increasing of the IOP. Daily fluctuations of IOP may be of the order of 10 mmHg, but changes in intraocular pressure do not have a noticeable effect on image quality [45]. The apparent contradiction can be justified with the presence in the eye of a compensating mechanism to correct the effects of the ocular dynamics on corneal shape and refractive status. This mechanism is necessary to reduce any potentially detrimental effects of IOP changes on the retinal image. Our model only considers the refractive power of the cornea associated to its purely mechanical response to the IOP.

An important effect of the corneal refractive surgery, which should receive more attention in the surgical practice, is the increase of the stress level inside the cornea. Quasi-static simulations provide a quantitative estimate of the stress distribution. The cornea is in a tensile stress state, and in preoperative conditions the highest stress is found in the optical zone. In the peripheral zone the stress decreases. The analysis of the contour levels of the NT component of the stress in Fig 14 shows clearly that the stress increases with the decreasing of the thickness of the cornea and reaches the maximum at the apex. In the postoperative case, this situation is magnified: the thinning of the central cornea due to the laser ablation causes a relevant increase of the local stress. The knowledge of the preoperative and postoperative stresses is instrumental in order to prevent the possible damage induced by the refractive surgery. As in the case of the apex displacement, the value of the stress in the central cornea assumes a particular relevance as indicator of possible damage. The anisotropic structure of the cornea suggests that the maximum, and therefore more significant, stress components should be found in the NT or SI direction. The NT and SI stresses at the 18 mmHg IOP for the five patients here considered are reported in Tables 5 and 6. While in the preoperative condition the maximum stress falls in the range 15.1–19.0 kPa, in the postoperative condition the stress grows to 18.4–26.8 kPa, with relative increments up to 52.5%. By comparing the data in Fig 16 and in Tables 3–5, one can observe that Patient III (Patient V), treated with maximum, 0.09 mm, (minimum, 0.04 mm) average ablation depth, shows the maximum, 28%, (minimum, 4%) postoperative increment of apical displacement and the maximum, 52%, (minimum, 18%) increment of the two NT and SI stress components in the anterior surface. All these observations confirm that thinner corneas are more sensitive to the IOP action.

The present study is limited in the sense that we did not explore the influence of different material models. Although the microscopic structure of the cornea is quite well understood, the identification of an accurate material model is still object of an intense research. However, an accurate material model without a patient-specific geometry will be unable to be predictive, therefore the contribution of this work can be considered as a step toward the creation of an authentic patient-specific model of the cornea.

Improvements of this work over our previous contribution [20] are: (i) the use of patient-specific geometrical models constructed from corneal topographer data, in contrast to an ideal model of the cornea; (ii) the adoption of a realistic material model that includes the dispersion of the collagen fibers, and (iii) a parametric analysis showing the quantitative changes in refractive and mechanical parameters following PRK interventions. Moreover, this work differs from [20] in the fact that here we are not simulating the corneal reprofiling [27], but only modelling the preoperative and postoperative geometry of the cornea. Future extensions of this work include the modelling of the variation of the fibril organization and interlacing across the cornea, with transition from the tridimensional organization of the fiber distribution to the planar observed in the innermost layers [35, 36]. Applications of the geometric patient-specific model here described include dynamic analysis to model the use of non contact tonometers (ORA and CORVIS), and the inclusion of intrastromal implants (ISR or INTACTS).

## Supporting Information

S1 Dataset Data for Patients in the preoperative and postoperative configurations. The.xyz files contain the coordinates lying on the anterior surface of th e cornea and.csv files contain the thicknesses associated to these points.
(ZIP)
